# Genome-wide association analysis of Mexican bread wheat landraces for resistance to yellow and stem rust

**DOI:** 10.1371/journal.pone.0246015

**Published:** 2021-01-29

**Authors:** Prashant Vikram, Deepmala Sehgal, Achala Sharma, Sridhar Bhavani, Priyanka Gupta, Mandeep Randhawa, Neftali Pardo, Daisy Basandra, Puja Srivastava, Sanjay Singh, Tanvi Sood, Carolina Paola Sansaloni, Hifzur Rahman, Sukhwinder Singh

**Affiliations:** 1 International Maize and Wheat Improvement Center (CIMMYT), El Batán, Texcoco, Mexico; 2 International Center for Biosaline Agriculture, Academic Ciy, Dubai, UAE; 3 Department Plant Breeding & Genetics, Punjab Agriculture University, Ludhiana, India; 4 International Center for Agricultural Research in the Dry Areas (ICARDA), Rabat Instituts, Rabat, Morocco; 5 CIMMYT—World Agroforestry Centre (ICRAF), Gigiri, Nairobi, Kenya; 6 Department Plant Breeding & Genetics, CSK HPKV Palampur, H.P. India; 7 ICAR-National Institute of Plant Biotechnology, Pusa, New Delhi, India; Institute of Genetics and Developmental Biology Chinese Academy of Sciences, CHINA

## Abstract

Deploying under-utilized landraces in wheat breeding has been advocated to accelerate genetic gains in current era of genomics assisted breeding. Mexican bread wheat landraces (Creole wheats) represent an important resource for the discovery of novel alleles including disease resistance. A core set of 1,098 Mexican landraces was subjected to multi-location testing for rust diseases in India, Mexico and Kenya. The landrace core set showed a continuous variation for yellow (YR) and stem rust (SR) disease severity. Principal component analysis differentiated Mexican landraces into three groups based on their respective collection sites. Linkage disequilibrium (LD) decay varied from 10 to 32 Mb across chromosomes with an averge of 23Mb across whole genome. Genome-wide association analysis revealed marker-trait associations for YR resistance in India and Mexico as well as for SR resistance in Kenya. In addition, significant additive-additive interaction effects were observed for both YR and SR resistance including genomic regions on chromosomes 1BL and 3BS, which co-locate with pleiotropic genes *Yr29/Lr46/Sr58/Pm39/Ltn2* and *Sr2/Yr30/Lr27*, respectively. Study reports novel genomic associations for YR (chromosomes 1AL, 2BS, and 3BL) and SR (chromosomes 2AL, 4DS, and 5DS). The novel findings in Creole wheat landraces can be efficiently utilized for the wheat genetic improvement.

## Introduction

The rust pathogens are ranked among the most ubiquitous fungal pathogens that continue to pose a serious threat to wheat production [1, 2]. Three rust diseases of wheat, namely; YR caused by *Puccinia striiformis f*. *sp*. *tritici* (Pst), leaf rust caused by *P*. *triticina* (Pt), and stem rust caused by *P*. *graminis f*. *sp*. *tritici* (Pgt) are of global significance [3]. Among them, stem rust is considered most devastating causing up to 100% yield losses in susceptible varieties. The recent emergence of highly virulent races of stem rust in the East African highlands combines unique and complex virulence defeating many resistance genes, which were previously effective against local stem rust races in individual geographies. The detection of a new and highly aggressive and complex stem rust race in Uganda in 1999 (also known as Ug99) has rendered more than 80% of global wheat varieties susceptible when tested in Kenya in 2008 [4]. The disease has migrated to over 13 countries across Africa, the Middle East, and West Asia. Over the past decade, 14 variants within the Ug99 race group have emerged and spread across East African countries [4, 5, Dave Hodson, CIMMYT, personal communication]. Recent localized epidemics of SR race not related to the Ug99 race group, called “Digelu race” (TKTTF), was reported in Bale region of southern Ethiopia [6]. The stem rust resistance in Digalu was postulated to be due to gene *SrTmp*. Although *SrTmp* was effective against some races in the Ug99 race group, the gene was ineffective against race TKTTF. The isolates of this race has also been reported in Turkey [7, 8], Lebanon, and Iran [4]. SR was not reported in Germany for decades before 2013 [9]. In 2013, SR infections were observed both in winter and spring wheat and race analyses found six Pgt races belonging to race TKTTF. The re-emergence of common barberry in Europe has led to stem rust epidemics in oats in Sweden [10]. Diverse races with various combinations of virulence have been identified from sexual populations in Georgia and Kazakhstan that can pose a threat to broader wheat production areas through long-distance dispersal, such as a recent epidemic of SR in durum wheat in Sicily caused by race TTRTF [11, 12].

In the current scenario, YR is considered as the most significant disease in major wheat growing countries around the world, largely due to its rapid evolution, spread and aggressive nature of new pathotypes. YR of wheat can cause up to 70% yield losses under severe epidemic conditions affecting the grain fill and quality. Annual losses of US $ 1 billion attributable to YR globally have been reported [13]. Murray and Brennan, [14] estimated average annual economic losses of AU $ 127 million caused by YR in Australia. YR has historically been endemic to areas with humid and cool summers or in warm high-altitude areas with cool nights [15, 16], but in recent years, YR has also been reported to show greater adaptation in warmer areas, where the disease was previously less important or practically absent [17]. In addition to temperature and moisture, YR epidemics are largely affected by growth stage and nutritional status of plants [18], host resistance characteristics [19, 20] the time of primary infection compared to crop growth [21], as well as the virulence characteristics of prevalent pathogen races [3, 22–26]. Repeated incursions of new races and/or entire population shifts at national or continental scales have been reported in several studies [27–32] and high rates of mutation from avirulence to virulence has been reported [33] which have contributed to increased susceptibility of varieties over area and time [34, 35].

Over 60 genes for SR and 83 genes for YR have been formally designated [36]. Among the 83 cataloged YR resistance genes [36, 37], most of them are race-specific genes except *Yr11*, *Yr12*, *Yr13*, *Yr14*, *Yr16*, *Yr18/Lr34/Sr57/Pm38/Ltn1*, *Yr29/Lr46/Sr58/Pm39/Ltn2*, *Yr30/Lr27/Sr2*, *Yr36*, *Yr39*, *Yr46/Lr67/Sr55/Pm46/Ltn3*, *Yr52*, *Yr59*, *Yr62*, *Yr68*, *Yr71*, *Yr75*, *Yr77*, *Yr78*, *Yr79*, *Yr80* and *Yr82* [38–40]. Five genes, namely *Sr2*, *Sr55/Yr46*, *Sr56*, *Sr57/Yr18*, and *Sr58/Yr29*, confer adult plant resistance showing enhanced pleiotropic resistance to other rusts [4]. It is often observed that combination 4–5 adult plant resistance genes with minor effects can provide near immune response against rusts. Sources of quantitative resistance in crop plants, readily detected in post-seedling growth stages and associated with non-race-specific resistance, have proven to be durable, making adult plant resistance (APR) a promising breeding target for long-term rust resistance [41, 42]. Therefore, wheat breeders must identify and characterize new sources of race-specific resistance and APR, and deploy into the popular high yielding but susceptible varieties. North- Western India including Punjab province is one of the hotspots for yellow rust disease.

Landraces are a valuable resource of trait diversity and offer an excellent choice for the incorporation of new traits into breeding germplasm pools as compared to wild relatives and other gene-bank accessions. Landraces and unadopted exotic germplasm stored in gene banks are a great reservoir of genetic variations that can be used to mitigate the current and future food challenges [8, 43–47]. Infusing breeding germplasm pool with landraces presents one way of broadening the breeding germplasm pool's genetic base, thereby enhancing the selection efficiency [48]. These accessions need to be included in a sizably large number in the breeding programs for achieving significant impact. The use of landraces to crop breeding has been practiced since long, although; on a limited scale [49]. The usual practice is to perform the phenotypic evaluation of landraces/ wild relatives, identification of extraordinary accessions and their introduction into the breeding pipeline. In addition, advances made in genotyping technologies led gene bank accessions to be genotyped at an unprecedented rate [50], however, these datasets can be utilized only in conjunction with phenotypic data.

Genome profiling is an essential tool for selecting important landraces for including them into the breeding pipeline(s). Broadly, there are two methods of germplasm identification based on genotype; one is through genomic assisted breeding value (GEBV) and other based on the presence/ absence and interaction of positive alleles. Wheat is a diversely adapted self-pollinated crop in which positive allele inclusion should be advantageous over GEBV based selection [51, 52]. Genome-wide association analyses provides a fast and effective way of mining positive alleles and their interactions, ultimately saving time and effort in the process. GWAS offers an enhanced number of variables subjected to analysis through using large number of accessions and markers as well for analysis [53, 54]. DArT-Seq offers a large number of genotypic variables that can be tested at a time in an experiment [53–56]. This study presents a case of GWAS in landrace core set of size 1,098 Mexican wheat landraces into the hotspot region (s) to identify new sources of resistance to YR and SR for an efficient deployment to the breeding programs.

## Materials and methods

### Plant material

The Mexican bread wheat landrace core set of 1,133 accessions, representing the complete breadth of variation of 7986 hexaploid landraces, was used for the study. Details of the complete set of Mexican bread wheat germplasm and representative core set are described in Vikram et al., [46]. This core set was formulated through using genotype and phenotype information simultaneously in a way to retain the maximum number of the rare alleles of the complete population of 7986 accessions. The core set of 1,133 genotypes represented phenotypic, genotypic, and geographical diversity of complete set of Creole wheat landraces as described by Vikram et al., [46].

### Yellow rust evaluations

Evaluation of the core reference sets for YR was performed under field conditions at CIMMYT’s experimental stations in Toluca, Mexico, and Punjab Agricultural University (PAU), India under field conditions. Evaluation at Punjab Agricultural University (PAU), India was performed during Oct 2015-Mar 2016, Oct 2016-Mar 2017 and Oct 2017-Mar 2018. Similarly, testing at Toluca, Mexico was conducted during May 2015-Oct 2015 and May 2016-Oct 2016. However, test results at Toluca, Mexico in 2016 (May 2016-Oct 2016) were not satisfactory, probably due to excessive rains and other field related factors, so this experiment results was was not used in genome- wide association analysis. Therefore, one season experiment at Toluca, Mexico and three-season experiments at PAU, India have been presented. All experiments were conducted with three replicates using an alpha-lattice field design.

At PAU, the core set of landraces were planted as two rows of 1m length, with row to row spacing of 22.5 cm along with cultivars namely, HD 3086, PBW 725 and Unnat PBW 343 as susceptible checks. Varieties PBW 343 and HD 2967 were used as an infector after every 20 rows and at the periphery of plots for the development of artificial epiphytotic conditions of YR. For field inoculations, urediniospores suspended in 10 liters of water with two drops of Tween 20, were sprayed at tillering and jointing stages using an ultra-low volume applicator on clear evenings with high expectation of dew to ensure proper disease infection. In addition, pots carrying infected plants were placed in the experimental field to create homogeneous epiphytotic conditions to avoid any disease escapes. A mixture of pathotypes of YR pathogen prevalent in the region (78S84, 110S119, 46S119 and 238S119) was used for inoculation [57] in 2015–16 as well as 2016–17 season. In 2017–18 season experiment, inoculation was carried out with slightly different pathotype mixture (78S84, 110S119, 46S119, 238S119 and XXS17). YR severity and response were recorded as percentage of the leaf area covered by a particular response according to the modified Cobb’s scale [58]. At Toluca, Mexico experiment was conducted in the rainy season (July- October); each genotype was sown in 0.7-m paired rows with 0.3-m pathway between rows in field trials. The YR spreader was mixed with susceptible wheat lines, such as Avocet/Attila cross, Morocco and Avocet near-isoline for gene *Yr31*. The spreader mixture was planted as hill plots in the middle of 0.3-m pathway and around the experimental area. The same Pst race (Mex14.191) as mentioned at seedling was sprayed onto stripe rust spreaders within and around test areas. The YR severity was assessed when Avocet showed 100% disease severity.

### Stem rust evaluation

The core set of 1,098 accessions were planted at the SR screening platform established at Kenya Agricultural and Livestock Research Organization (KALRO), Njoro for evaluation against SR race Ug99 and its derivatives during the main seasons of 2015 and 2016. Bread wheat line ‘Cacuke’ was used as a susceptible check. Landraces and susceptible check ‘Cacuke’ were sown as completely randomized design with two replicates. Genotypes were planted twin-row plots of 0.7m length with 20 cm space between rows and 0.3-m pathway. Five-gram seeds of each genotype carrying about 60–70 seeds were sown in each plot. Infectors comprised of a mixture of SR susceptible *Sr24* carrying lines (GIDs: 5391050, 5391052, 5391056, 5391057, 5391059, and 5391061) and cultivars ‘Cacuke’ and ‘Robin’ were planted as hill plots on one side of each experimental plot in the middle of the 0.3 m-wide pathways. Infectors were also planted along the borders of the experimental field in 1 m plots to facilitate uniform disease build-up and spread. At early booting stage, freshly collected urediniospores of SR races of Ug99 race group (TTKSK, TTKST and TTKTT) suspended in distilled water, were injected into culms in the infector plots by using a hypodermic syringe over at least three occasions. Disease severity and the response were recorded on two occasions; first, when susceptible check Cacuke displayed 50–60% of disease severity and second when it showed 100% SR severity (at dough stage of plant growth). Disease severity and response were recorded using the modified Cobb Scale [58]. The final disease severity score was used for analysis.

### Genotype data analysis and haplotype characterization

From an initial set of 11,230 SNPs on 1,133 landraces, a filtered set of 6,917 SNPs was obtained after eliminating unmapped markers and culling markers with >30% missing data and MAF<0.05. Also, landraces with missing data of more than 60% were also removed. A final dataset of 6,917 SNPs on 1,098 landrace accessions was used for all analysis. All SNP alleles of each genotype were transformed to a number; the bases A, C, G and T were changed to 1, 2, 3 and 4, respectively. A zero (0) was assigned to all SNP with missing data. Haplotypes were generated in R according to the algorithm described by [59]. Hardy Weinberg p-value cut off was set to 0.001, and the minimum marker allele frequency was set to 0.05. Individuals with more than 75% missing data were excluded. The resulting haplotypes were displayed as blocks of marker numbers and alleles [48]. Two season evaluation data sets of YR (2015–16 and 2016–17) and SR (Kenya 1 and 2) were used for the genome-wide analysis. The third season (2017–18) YR data was not used for genetic analysis due to a slight difference in the pathotypes used for inoculation.

### Linkage Disequilibrium (LD) and GWAS analysis for marker-trait associations

The squared correlation coefficient (*r*^*2*^), a measure of LD, was estimated among all possible pairs of markers using GAPIT version 2.0 [60] and the pattern of LD decay was visualized by plotting pair-wise r^2^ values against the physical distance (Mb). A smooth line was fit to the data using second-degree locally weighted scatterplot smoothing, LOESS [61] as implemented in SAS. For the LOESS estimation of LD decay, genetic distance was estimated as the point where the LOESS curve first crosses the baseline *r*^*2*^ of 0.1.

The covariance matrix was derived by PCA using the function PRCOMP from the STATS package in R. The kinship matrix was calculated by the VanRaden algorithm. A mixed linear model was used in which PCA is used as a fixed variate and kinship as random. The numbers of PCs used as a fixed variate were determined using the Bayesian Information Criterion (BIC; [62]). GWAS analysis was conducted in Plink version 1.07 executed in R software [63]. The information on chromosome arm was obtained by performing BLAST of the sequences in the Ensemble Plants database (https://plants.ensembl.org/Triticum_aestivum/Info/Index).

## Results

### Rust evaluation

**Fig 1:**
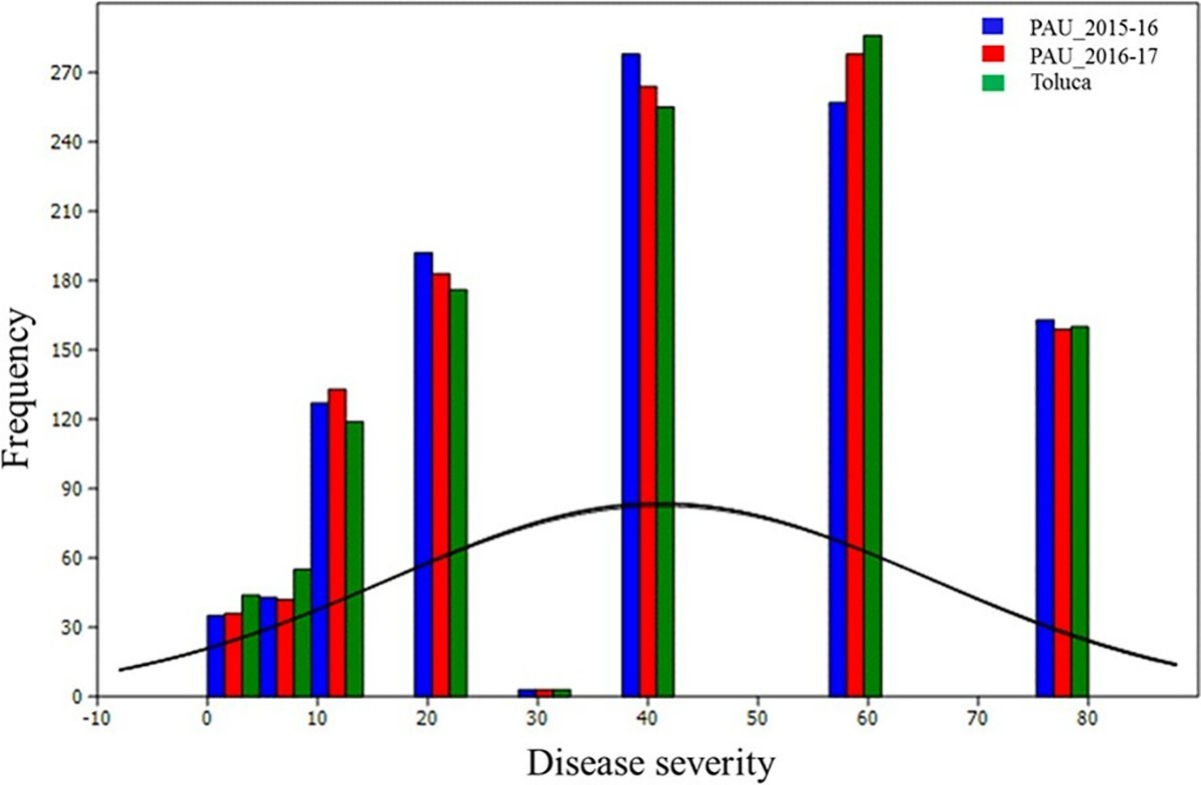
Graph showing the frequency distribution of Mexican bread wheat landraces for Yellow Rust (YR) disease severity score in India (PAU: Punjab Agriculture University, 2015–16 and 2016–17 seasons) and Mexico (Toluca: 2016–17 season). Disease severity score plotted on X-axis and frequency on Y-axis, respectively.

**Fig 2:**
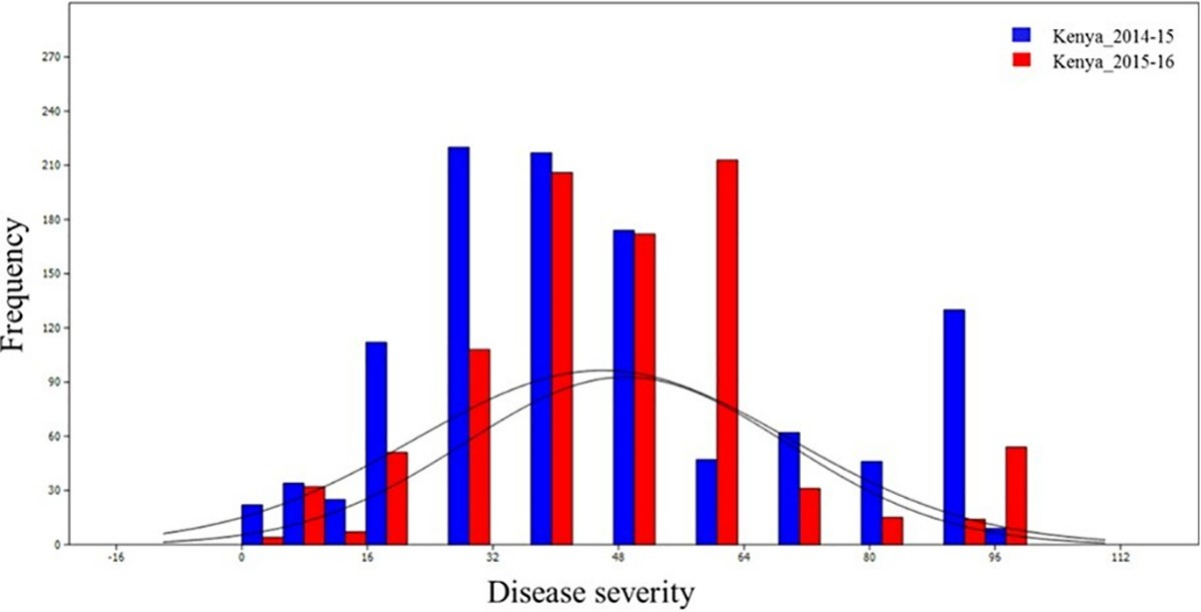
Graph showing the frequency distribution of Mexican bread wheat landraces for Stem Rust (SR) disease severity score in Kenya during 2014–15 and 2015–16. Disease severity score plotted on X-axis and frequency on Y-axis, respectively.

The Mexican landrace core set reflected substantial variation for YR and SR severity. Frequency distributions for YR and SR disease severity are presented in Figs 1 and 2, respectively. The frequency distribution showed continuous variation for YR and SR disease severity in India and Mexico. YR severity ranged from 0 to 80% and genotype frequency from 5 to 400 over three test environments. The YR disease severity ranged from 3.5 to 75.0%, 6.0 to 77.0%, 5.5 to 85% and 7.0 to 80.0% at PAU 2015–16, PAU 2016–17, PAU 2017–18 and Toluca, respectively. The Mexican landraces showed wide distribution for SR disease severity values in Kenya. The SR disease severity values ranged from 0.0 to 96.0% and 5.0 to 100.0% during main seasons 2015 and 2016 at Njoro, Kenya, respectively.

**Fig 3:**
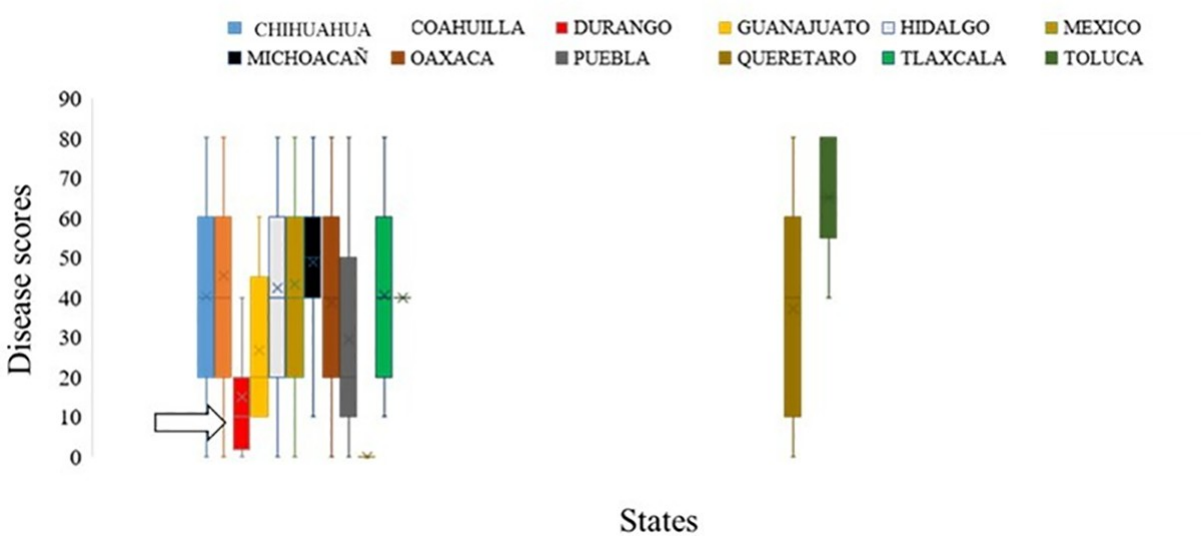
Figure presenting disease severity of Mexican wheat landraces belonging to different states against yellow rust fungus. Accessions from Durango showed the lowest disease score as compared to the ones from other states. X-axis presents states and disease score was plotted on Y-axis.

**Fig 4:**
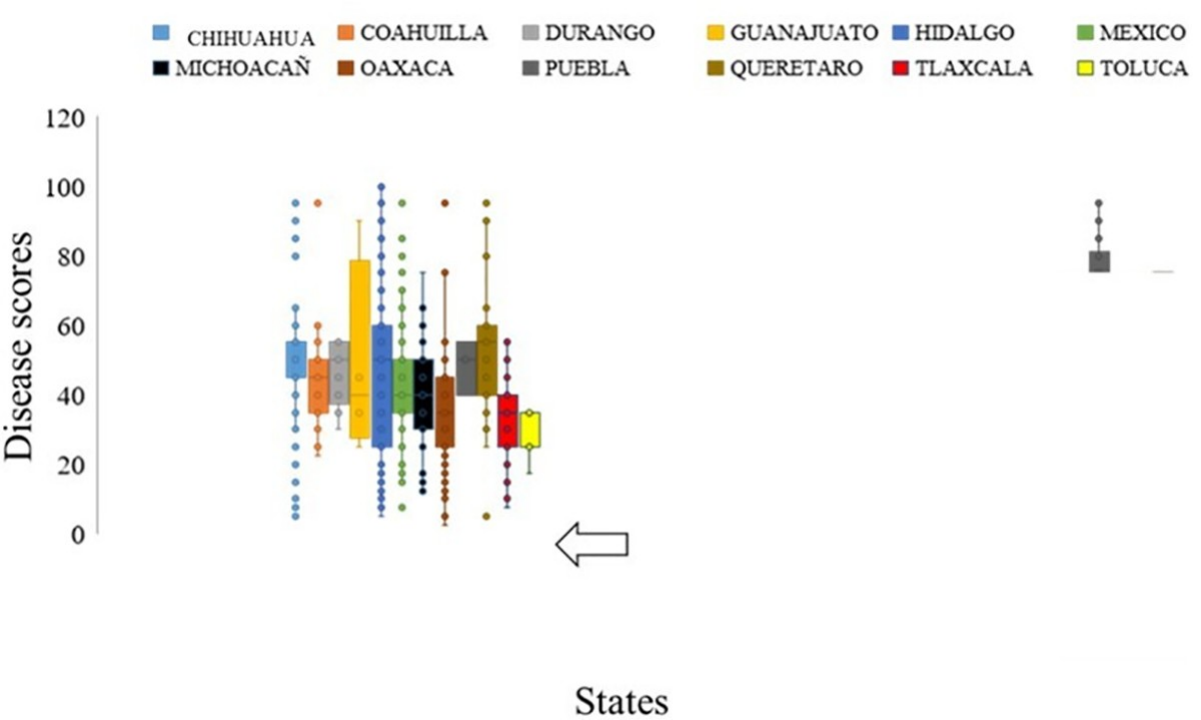
Figure presenting disease severity of Mexican Wheat landraces belonging to different states against stem rust fungus. Accessions from ‘Tlaxcala and Toluca’ showed lowest disease score as compared to the ones from other states. X-axis presents states and disease score was plotted on Y-axis.

Spatial analysis of the Mexican landraces revealed that accessions from ‘Durango’ state of Mexico showed moderate to high resistance against YR fungus at PAU, Ludhiana, India. Similarly, landraces belonging to ‘Tlaxcala’ and ‘Toluca’ states revealed their importance for imparting SR resistance in wheat cultivars (Figs 3 and 4). A significant and high correlation was observed between YR disease severity of genotypes at PAU in three consecutive season experiments. Between 2015–16 and 2016–17 season Pearson’s correlation coefficient was *r*^*2*^ = 0.97. Between 2015–16 and 2017–18 season trials it (r^2^) was 0.96; similarly, between 2016–17 and 2017–18 season testing the figure (r^2^) was 0.98. The correlations between PAU and Toluca environments were extremely low and not considered significant (S1 Table), suggesting different resistance loci are involved in resistance to YR in two environments. A moderate but significant correlation (0.62) was observed between SR disease severity in two main seasons at Kenya in years 2015 and 2016.

### Genetic analysis

**Fig 5:**
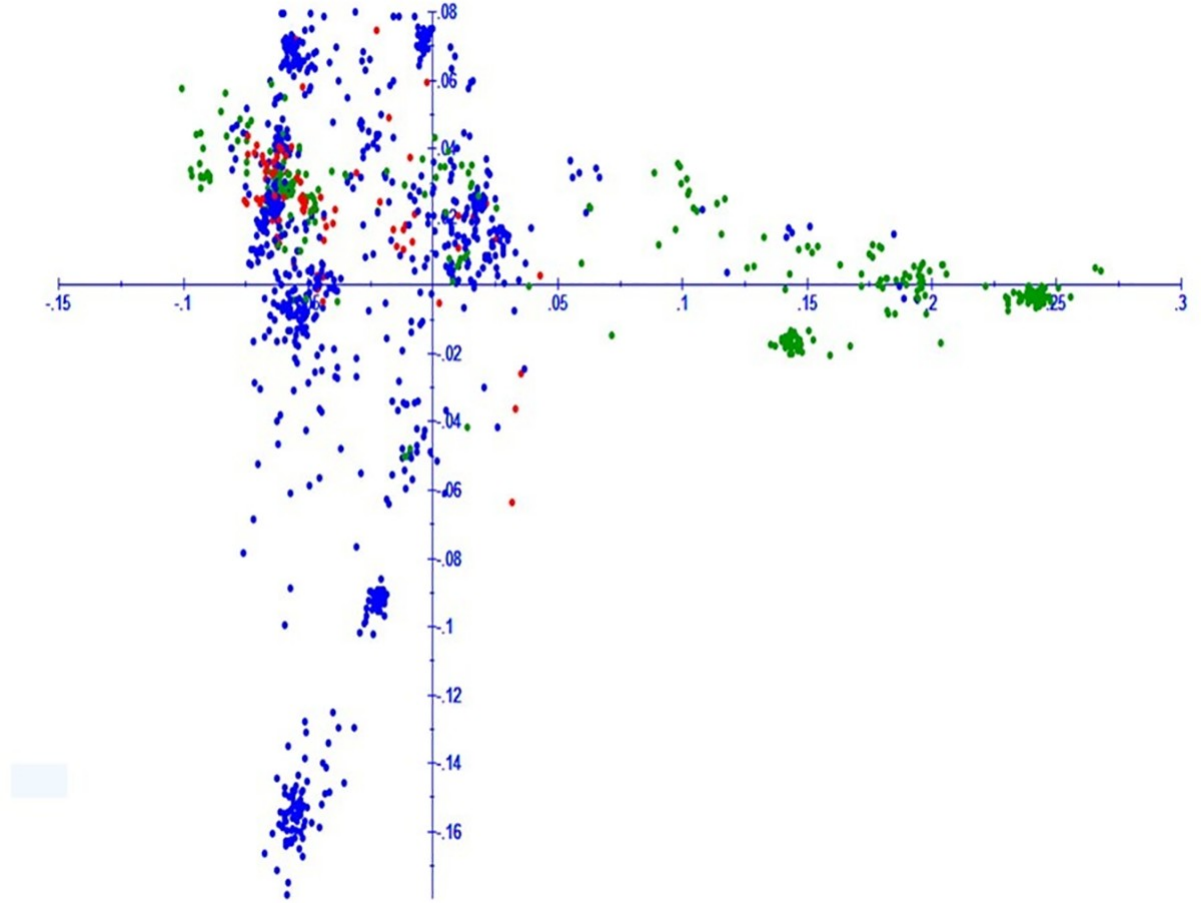
Two dimensional PCA plot sowing grouping of Mexican landraces from three regions determined through DArT-seq SNP markers. Landrace accessions belonging to northern, central and southern Mexican states are depicted as green, blue and red dots, respectively, in the graph.

**Fig 6:**
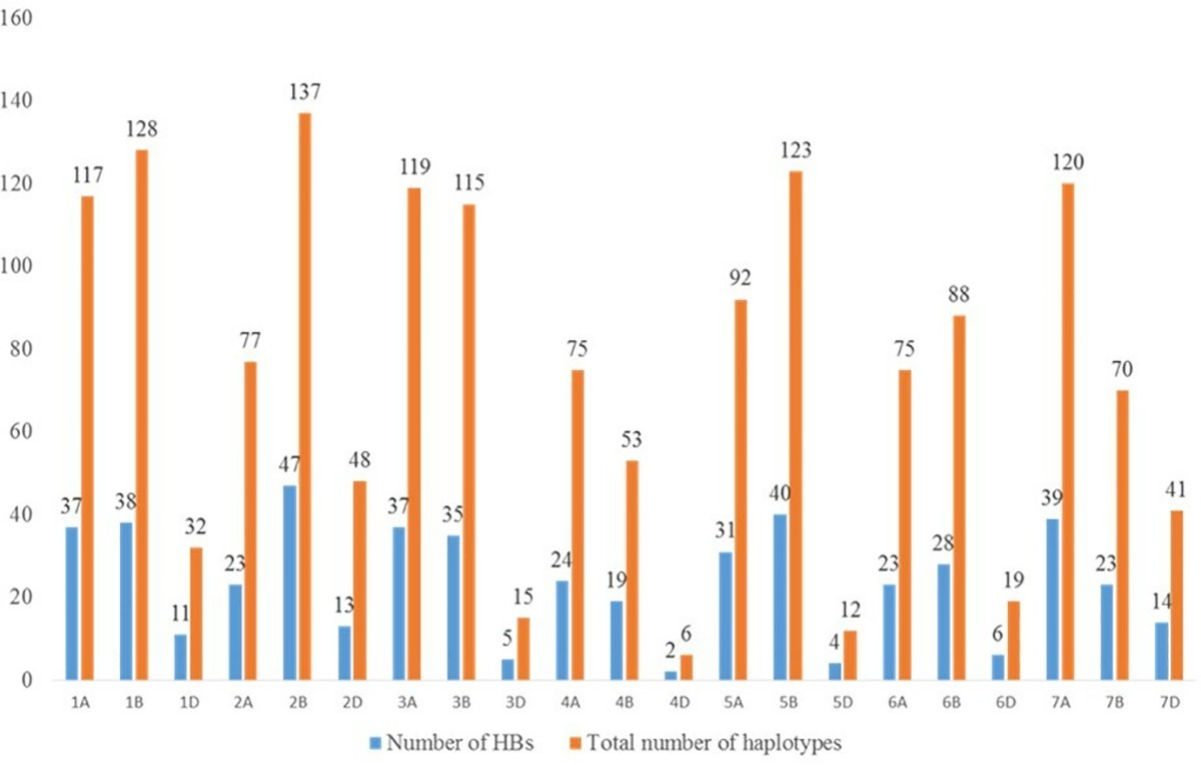
Genome-wide distribution of haplotype blocks and haplotypes. Graph confirms that number of haplotype blocks and haplotypes are highest in ‘B’ genome followed by ‘A’ and least in ‘D’.

PCA revealed three clusters harboring landraces from central, northern and Southern Mexico comprising 704, 306 and 86 accessions, respectively. Most of the accessions from north Mexico were found distinct from those belonging to Central and South Mexico, while the South group was strongly associated with the Central group (Fig 5). Haplotype block (HB) analysis revealed 1,562 haplotypes corresponding to 499 genome-wide HBs. Around 214, 230 and 55 HBs were specific to A, B and D genomes, respectively. Number of haplotypes on A, B and D genomes were 675, 714 and 173, respectively. The number of haplotype blocks on chromosomes ranged 6 to 137. The number of SNPs in HBs ranged 2 to 47 (Fig 6). The average genetic distance at which LD across all chromosomes decayed (*r*^*2*^ < 0.1) was found to be in range of 10 to 32Mb (S2 Table). The LD decay of 10–32Mb indicated that these landraces were genetically diverse.

### Genomic associations for yellow rust and stem rust resistance

**Fig 7:**
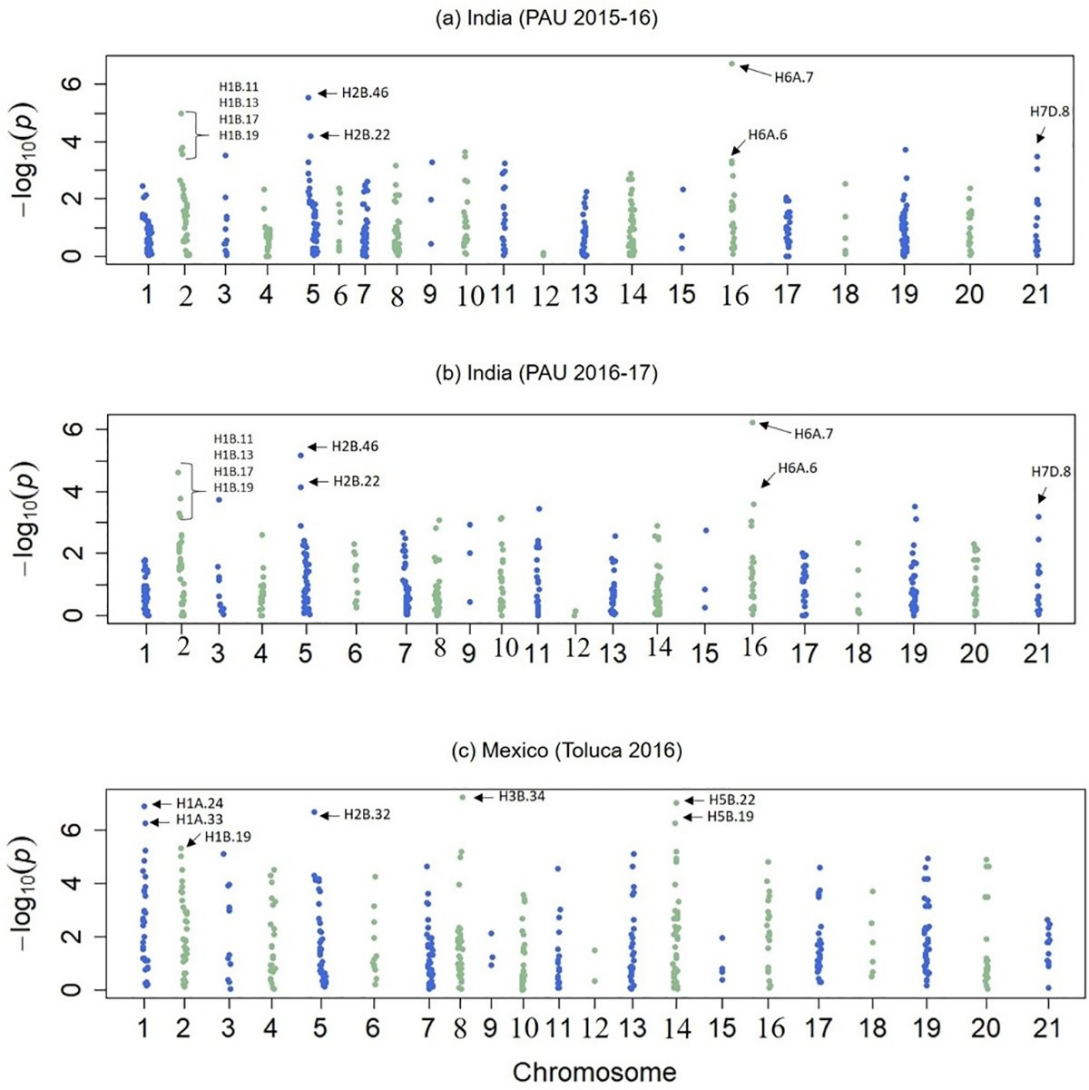
Manhattan plots presenting significance of haplotype blocks with YR resistance in the Mexican wheat landrace core set population in India (two seasons in Punjab Agriculture University, Ludhiana) and one season in Toluca, Mexico. Significance (-Log_10_P) is presented on Y-axis and chromosomes on X-axis. Chromosome numbers 1 to 21 correspond 1A to 7D respectively.

**Fig 8:**
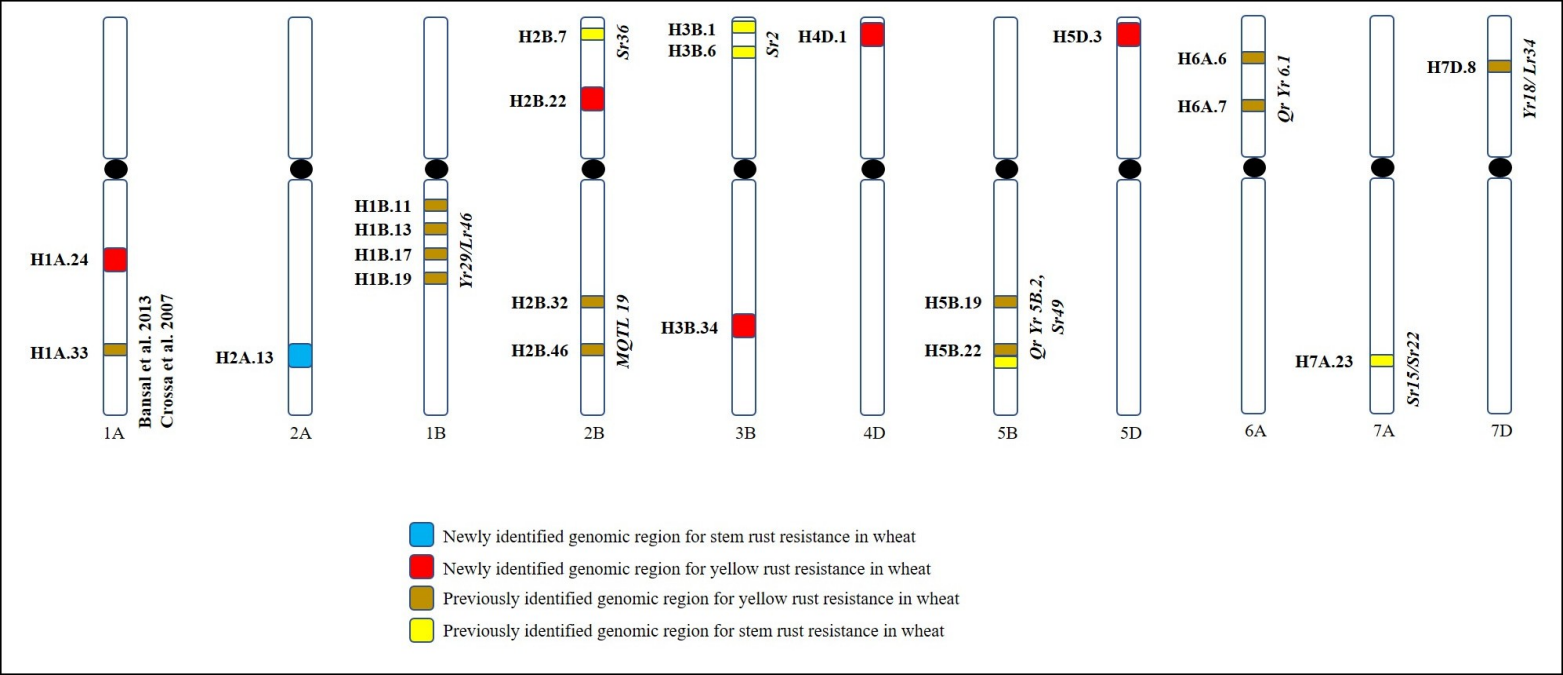
Figure presenting location of haplotype block–trait association on wheat hromosoe arms. Novel and previously identified genomic regions have been pictorially depicted in the figure.

For YR resistance, nine HBs were identified on chromosomes 1BL (4), 2BS (1), 2BL (1), 6AS (2) and 7DS (1) in PAU environments. QTL clusters were identified on chromosomes 1BL, 2BL and 6AS where multiple HBs and/or SNPs were identified associated with YR resistance loci (Fig 7, Table 1, S3 Table). QTLs overlapping with previously reported genomic regions and novel QTL regions are presented in Fig 8. Four HBs colocating with well known *Yr29/Lr46* gene i.e. H1B.11, H1B.13, H1B.17 and H1B.19 were identified on chromosome 1BS that reduced disease severity from 7.7 to 18.6% in two seasons at PAU (Table 1). Five SNPs (GBS tags- 2276098, 1120791, 1090342, 984340 and 999620) which are a part of these HBs were found significantly associated with YR resistance in single marker GWAS (S3 Table). Similarly, two adjacent HBs were identified on chromosome 6AS *viz*. H6A.6 and H6A.7, that reduced disease severity by 10.8–11.9% and 18.1–19.2%, respectively. The *Yr18/Lr34*-linked locus H7D.8 on chromosome 7DS, showed minor effects on YR resistance in this panel. In Toluca environment, seven HBs on chromosomes 1AL (2), 1BL (1), 2BL (1), 3BL (1) and 5BL (2) were identified to be associated with YR resistance with QTL clusters identified on chromosomes 1AL and 5BL (Table 1). On chromosome 1AL, two HBs i.e. HB1A.24 and HB1A.33 reduced disease severity by 16.1 and 20.0%, respectively, whereas HBs on chromosome 5BL (HB5B.19 and HB5B.22) showed reductions by 10.2 and 21.4%, respectively. The *Yr49/Lr46*-linked locus HB1B.19 showed almost similar effects in reducing disease severity at both PAU and Toluca environments.

**Table 1:** Important haplotype associations with yellow and stem rust resistance.

Trait/environment	Hap. block	Chro moso me	Haplotype alleles	Favorabl e allele	Reduction in severity (%; min—max)	Overlap with known genes, QTL or MetaQTL
YR resistance/Toluca	HB1A.24	1AL	CCA, CCG, CTG, GCG	CCA	16.1	***New***
	HB1A.33	1AL	AC, GC, GT	AC	21.0	*Bansal et al*. *2013; Crossa et al*. *2007*
	HB1B.19	1BL	CC, GC, GG	CC	10.0	*Lr46/Yr29*
	HB2B.32	2BL	AC, AT, GT	AC	12.8	*MQTL19*
	HB3B.34	3BL	AA, GA, GG	AA	21.7	***New***
	HB5B.19	5BL	CA, CG, TG	CA	10.2	*QRYr5B*.*2*
	HB5B.22	5BL	CAT, CGC, GGT	CGC	21.4	*QRYr5B*.*2*
YR resistance/PAU	H1B.11	1BL	CC, TC, TT	TT	11.2–12.0	*Yr29/Lr46*
	H1B.13	1BL	CA, TA, TG	TA	7.6–7.7	*Yr29/Lr46*
	H1B.17	1BL	AA, AT, TA	TA	18.0–18.6	*Yr29/Lr46*
	H1B.19	1BL	CC, GC, GG	CC	9.4–9.9	*Yr29/Lr46*
	H2B.22	2BS	AC, CC, CG	CG	9.0–9.6	*New*
	H2B.46	2BL	AC, GC, GT	AC	9.1–14.3	*MQTL19*
	H6A.6	6AS	CG, TA, TG	CG	10.8–11.9	*QRYr6A*.*1*
	H6A.7	6AS	CC, CT, TC	CT	18.1–19.2	*QRYr6A*.*1*
	H7D.8	7DS	CG, CT,	GG	6.3–6.6	*Yr18/Lr34*
SR/Kenya	H2A.13	2AL	CA, CT, TT	CA	19. 7–20.1	***New***
	H2B.7	2BS	AA, AG, GA	AA, GA	21.9–24.6	*Sr36 or Sr40*
	H3B.1	3BS	CG, GA, GG	CG, GG	16.0–16.9	*Sr2*
	H3B.6	3BS	CA, CT, GT	CT, GT	15.8–22.8	*Sr2*
	H4D.1	4DS	AA, GA, GT	GT	22.7–25.4	***New***
	H5B.22	5BL	CAT, CGC, GGT	CGC, GGT	13.8–29.2	*Sr49*
	H5D.3	5DS	CC, CT, TC	TC	23.9–25.8	***New***
	H7A.23	7AL	CA, TA, TC	CA	6.7–12.7	*Sr15 or Sr22*

**Fig 9:**
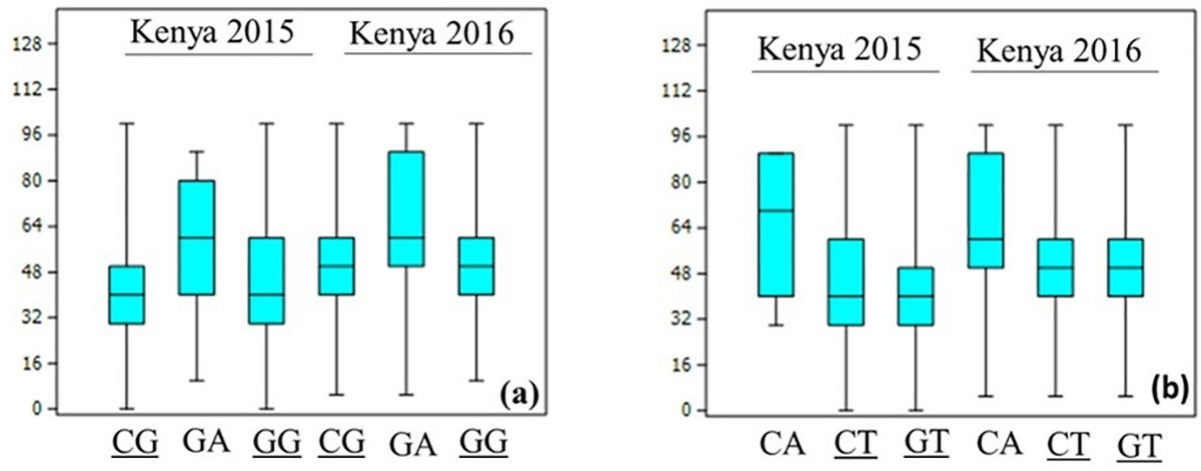
Effect of Sr2- linked haplotypes H3B.1 (a) and H3B.6 (b) on SR resistance in the two seasons at Kenya. Two favorable haplotypes identified at each locus are shown as underscored.

Eight HBs were significantly associated with SR resistance in both test environments (Kenya 2015, Kenya 2016). These associations were identified on chromosomes 2AL (1), 2BS (1), 3BS (2), 4DS (1), 5BL (1), 5DS (1) and 7AL (1). The most significant associations were identified on chromosomes 2B, 4D and 5D where HBs showed more than 20% reduction in disease severity in both seasons at Kenya (Table 1, S4 Table). The two loci, H3B.1 and H3B.6 reduced disease severity by 16.0–16.9% and 15.8–22.8% in 2015 and 2016, respectively. Two favorable alleles were identified at both these loci, (Fig 9).

**Fig 10:**
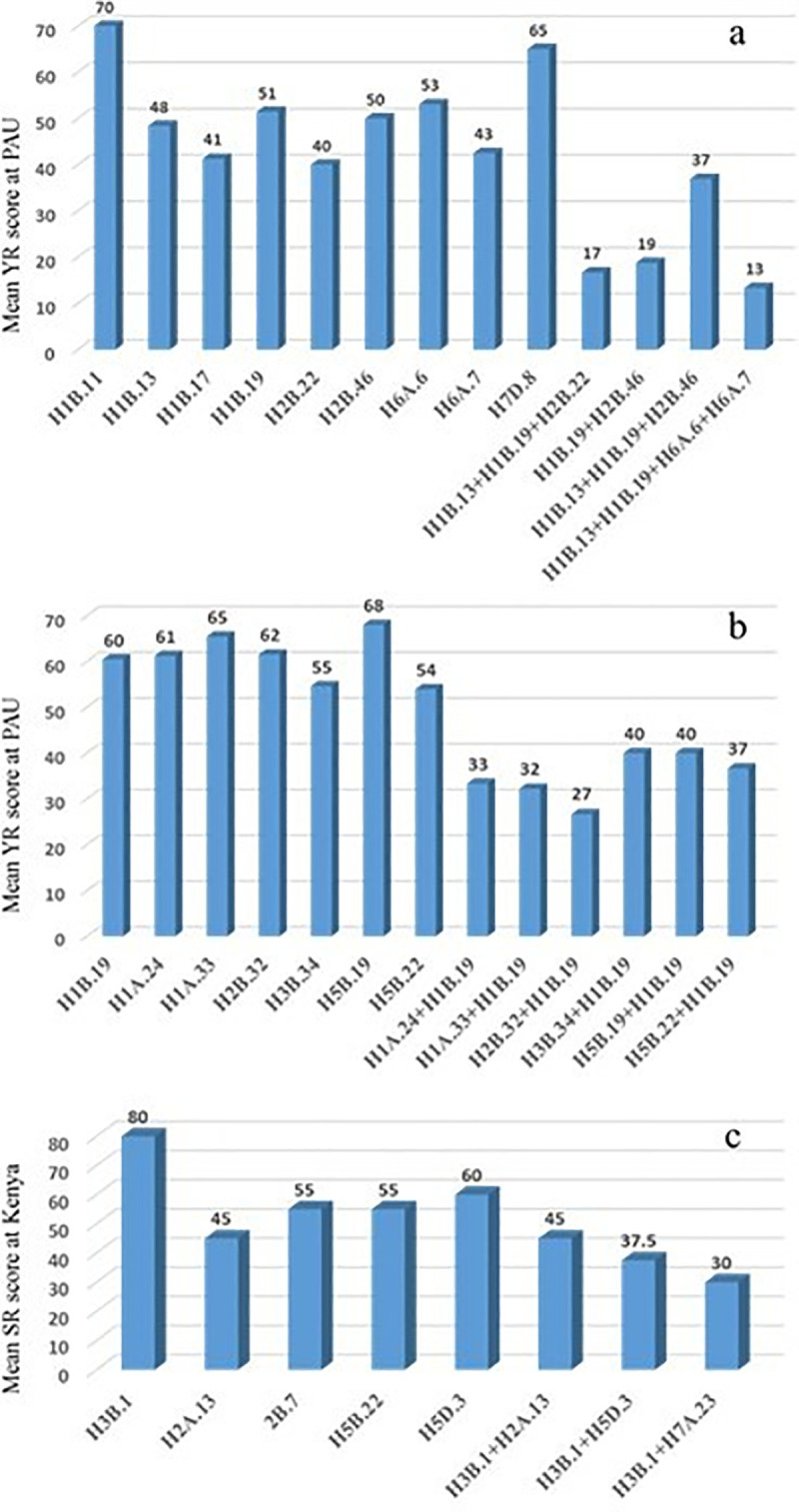
Individual QTL-QTL combination effects on mean YR at PAU (a) and Toluca (b) environments and on mean SR resistance (c) at Kenya. Haplotype(s) H1B.19 or a combination of H1B.13 and H1B.19 was used to represent additive QTL effects for YR resistance- associated loci, whereas haplotype H3B.1 is used to show additive effects for SR resistance-associated loci.

We further dissected the effects of the identified QTL individually or as additive effects in two- QTL combinations. In the latter case, HBs in the *Yr49/Lr46* and *Sr2* genomic regions were kept fixed (Fig 10). For PAU environments, additive effects of YR loci were presented as combinations of HB1B.19 or HB1B.13+HB1B.19 with other QTL. Since, H3B.1 and H3B.6 provided similar results for SR resistance, we present the additive interaction only with H3B.1. The mean YR severity was numerically lowest with three combinations in PAU environment i.e. H1B.13+H1B.19+H2B.22, H1B.19+H2B.46 and H1B.13+H1B.19+H6A.6+H6A.7 (Fig 10A), whereas it was lowest with four combinations in Toluca i.e. H2B.32+H1B.19, H1A.33+H1B.19, H1A.24+H1B.19 and H5B.22+H1B.19 (Fig 10B).

The mean SR severity was lowest with two combinations; H3B.1+H7A.23 and H3B.1+H5D.3 (Fig 10C).

The–Log10 (p-value) is plotted on Y-axis. SMA-PAU_15–16, SMA-PAU_16–17 and SMA- Toluca correspond to single-marker associations with first and second season evaluation at Punjab Agriculture University, India and one season evaluation at Toluca, Mexico datasets respectively. Similarly, HB associations with these datasets have been indicated with HB-PAU_15–16, HB-PAU_16–17 and HB-Toluca. Two genomic regions harboring four or more haplotypes on chromosomes 1B, 3A and 6A are presented with dotted lines.

### Interaction effects among haplotype blocks

**Fig 11:**
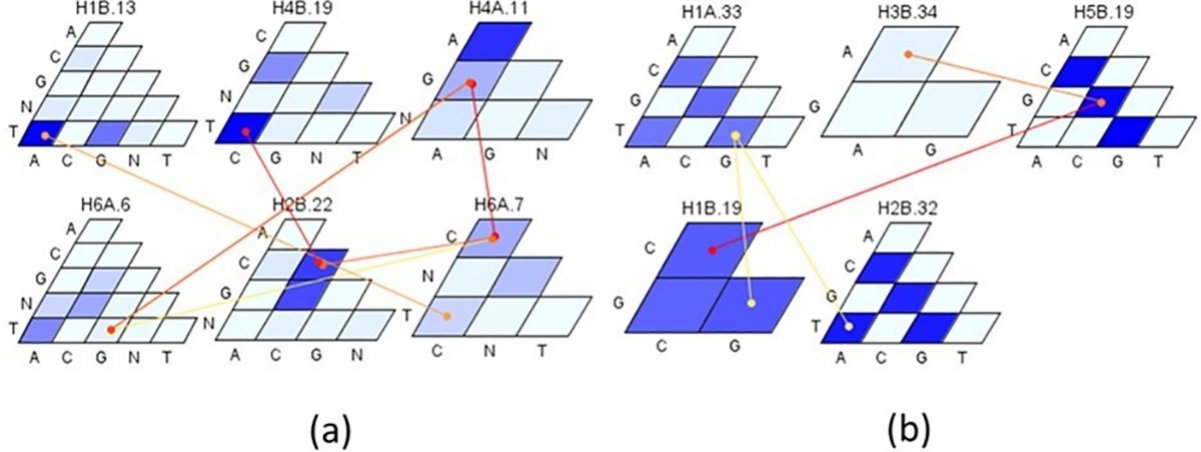
Additive-Additive interactions among haplotypes for yellow rust disease in PAU (a) and Toluca (b) environments. The magnitude/effect of interactions is presented with yellow and red colors where red corresponds to stronger interaction at P <0.001.

**Fig 12:**
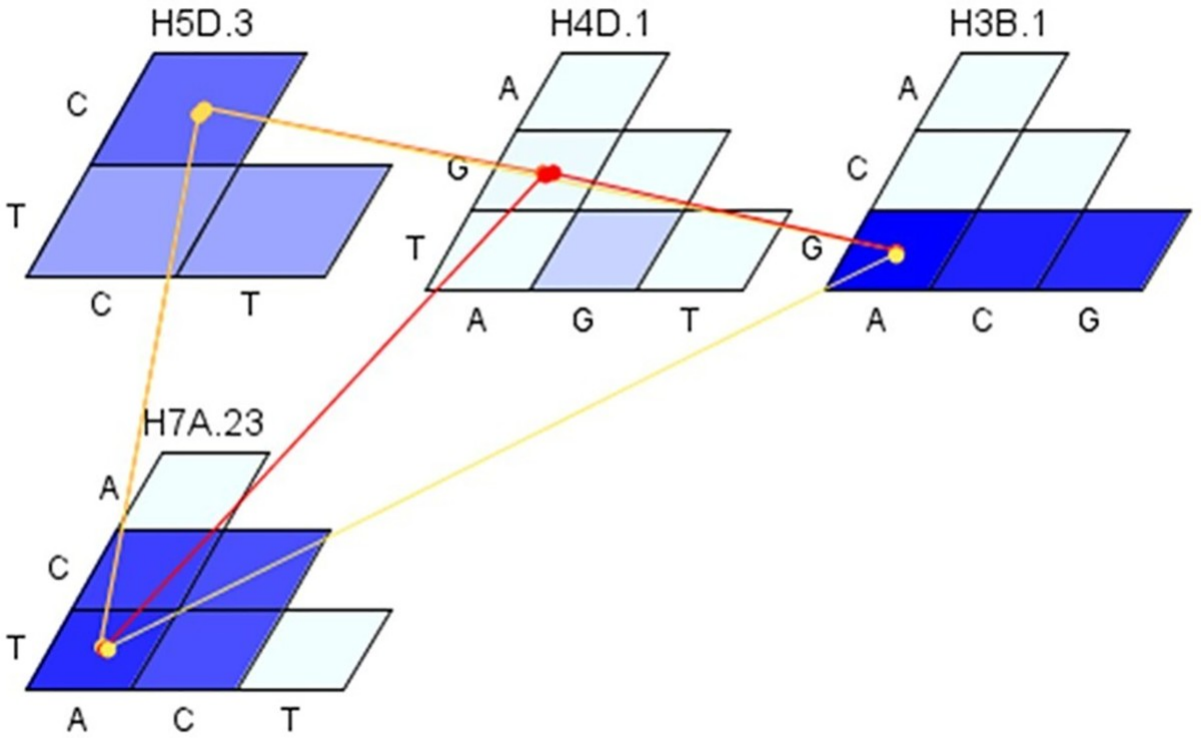
Additive-Additive interactions among loci associated with SR resistance. The magnitude/effect of interaction is presented with colors from yellow to red (stronger interaction). The haplotype H3B.1 is significantly involved in the Additive-Additive interaction effect with haplotypes H4D.1 and H7A.3.

For YR resistance, strong additive-additive interactions were observed among six loci in PAU and among five loci in Toluca (Fig 11). The HBs on chromosome 1B collocating *Yr29/Lr46* gene(s) were significantly involved in the interaction in both environments i.e. H1B.13 in PAU and H1B.19 in Toluca. Additionally, in PAU, interactions of H6A.7 and H2B.22 were observed with other loci contributing to an additional variation of 6.2%. In Toluca environment, additional interactions were observed between H1A.33 and H2B.32 and between H5B.19 and H3B.34 (Fig 11). For SR resistance, four HBs were significantly involved in additive-additive interactions (Fig 12). The H3B.1 locus showed significant interactions with haplotypes H4D.1 and H7A.3 with an average percentage variation of 7.5%. In addition, a complex network of interactions was observed among haplotypes H5D.3, H4D.1 and H7A.23 contributing to an additional variation of 6.2%.

## Discussion

Wheat landraces harbour numerous traits that are relevant for enhancing productivity and resilience. The inclusion of diverse landraces can significantly help in broadening the genetic base of breeding germplasm pools. However, landraces have been exploited modestly by breeders for trait enrichment primarily due to their unadaptable phenotypic traits, linkage drag, and low yield potential as compared to elite cultivars. Mexican bread wheat landraces have shown a moderate to the high level of YR and/or SR resistance under field evaluations in Mexico [46]. Pre-breeding germplasms derived from these landraces also showed resistance to YR [48]. Mexican landrace derived lines are being evaluated in the varietal pipeline at PAU, India (Achla Sharma, personal communication).

In this study, the Mexican bread wheat landrace core set was evaluated for YR and SR at different locations. This germplasm set reflected substantial variation for YR and SR resistance. The YR reaction of genotypes at PAU 2015–16 and 2016–17 showed significant positive correlation (*r*^*2*^ = 0.97). It is interesting to note that the YR reactions of genotypes at Toluca, Mexico, had no significant positive correlations with PAU, indicating different resistance genes controlling YR resistance at Toluca, Mexico and PAU, India which is also explained due to pathogen diversity in the two different geographies. The correlation between disease response of landrace accessions against SR in two seasons at Kenya (2015 and 2016) was moderate (0.62) but significantly correlated. Further, the frequency distribution of the landrace population revealed the normal distribution of both YR and SR scores, rendering it a fit candidate for mapping YR and SR resistance genes. Genome-wide association using a ‘HB-trait analysis’ approach was performed to understand the genetic basis of YR/SR resistance of landraces evaluated in the study. This approach is an efficient method for identifying genomic associations [64, 65]. The HBs offer a promise of identifying genomic associations with higher confidence as compared to the single markers. The HBs harbour two or more independent SNPs belonging to the same chromosomes, and therefore two or more independent variables are tested at the same time for the trait associations. A total of 499 HBs having 1,562 haplotypes with 2 to 47 SNPs in each HB were analysed. B genome had the highest number of HBs (230) followed by the A (214) and D (55) genomes. The D genome had minimum number of markers and therefore, had minimum possibility of recombination, leading to a smaller number of HBs as compared to A and B genomes (Fig 4). These results are in accordance with the genome size of cultivated wheat [66] and indicate genomic relatedness of elite bread wheat and landraces. Further, linkage disequilibrium analysis revealed the LD decay of 10 – 32Mb across chromosomes with genome wide decay at 23Mb in the Mexican bread wheat landrace core set in this study). Previous studies have reported a slower LD decay of 10–40 cM in the elite bread wheat populations which corresponds to ~ 30 – 120Mb [67–72]. Genome wide LD decay was observed at 23 Mb in the whole panel which is in the same range as reported recently in a CIMMYT spring wheat collection [67]. Principal component analysis broadly grouped landraces based on their collection site information (south, north, and central Mexico).

To investigate the novelty of associations obtained in the present study, we compared their locations with previously reported *Yr* and *Sr* genes and/or QTL reported in GWAS studies. In addition, consensus maps reporting meta-QTL (MQTL) for YR and SR resistance were also used to explore overlaps, if any [73–75]. Several significantly associated loci coincided with the presence of known major genes or QTL for rust resistance. On chromosomes 1B and 2B, multiple HBs were detected associated with YR resistance in both environments (PAU and Toluca). On chromosome 1BL, associations were obtained where an APR gene *Yr29/Lr46* has been mapped. Based on physical positions, these HBs were within 9–20 Mb of *Yr29/Lr46* gene. On chromosome 2B, we obtained three HBs associated with YR resistance; H2B.22 on short arm (PAU) and H2B.32 (Toluca) and H2B.46 (PAU) on the long arm. All three HBs on chromosome 2B showed minor effects and are suspected to be APR QTL (Table 1). YR gene *Yr27* has been mapped to chromosome 2BS, three meta-QTL have been reported on the same chromosome [74]. *Yr27* is ineffective to both Mexican and PAU pathotypes, and the physical distance between H2B.22 and three meta-QTL is from 219–269 Mb. H2B.22, and the phenotypic effect is minor; therefore, could be a new QTL. On 2BL, five all-stage resistance genes (*Yr5*, *Yr44*, *Yr53*, *Yr43* and *Yr3*) have been mapped and no APR gene has been reported. Of the three meta-QTL located on 2BL [73], both H2B.32 and H2B.46 overlapped with M-QTL19 [73]; this region needs further characterization to confirm the gene identity.

In the Toluca environment, two associated HBs on chromosome 1AL were about 60 Mb apart based on physical distance, indicating the possibility of two different resistance loci for YR resistance. On chromosome 1AL no YR gene has been reported; however, MTAs on chromosome 1AL has been detected in GWAS studies in Watkins collection and in CIMMYT’s historical germplasm [70, 76]. Comparison of physical distance among 1AL QTL reported in published studies revealed that H1A.33 was more linked (within 16 Mb) to 1AL QTL of Bansal et al., [76] and Crossa et al., [70]. H1A.24 was 34 Mb proximal to this QTL and could be a new QTL at that locus. Two more associations that were explicitly identified in Toluca were on chromosomes 3BL and 5BL. On chromosome 3BL, the only YR resistance locus is *Yr80* which is located 208 Mb proximal to H3B.34. The two meta-QTL reported on 3BL are on an average 74 Mb away. Hence, H3B.34 could be a new QTL. On chromosome 5BL, two linked HBs (H5B.19 and H5B.22) were associated with YR resistance. Meta-QTL *QRYr5B*.*2* reported by Rosewarne et al., [74] was within 20 Mb from these HBs. Crossa et al., [70] reported a QTL on 5BL in CIMMYT germplasm, 164 Mb, distal to HBs 5B.19 and 5B.22. In PAU environment, the two HBs identified on 6AS overlapped with meta-QTL *QRYr6A*.*1* of Rosewarne et al. [74] which is close to the telomeric region. Associations on chromosome 6AS have previously been reported in CIMMYT germplasm [70] which physically are 17 to 36 Mb from H6A.6 and H6A.7 reported here. The H7D.8 showing minor effects on YR resistance in PAU environment was intricately linked to *Yr18/Lr34* gene.

For SR resistance, H2A.13 is likely a new SR resistance locus because the only known gene in the region, *Sr21*, is ineffective against *Ug99* race group. Recently, a minor effect gene on chromosome 2AL was also identified in biparental populations investigated for SR resistance at CIMMYT, thus confirming it to be a new locus (Sridhar Bhavani, personal communication). H2B.7 was identified on chromosome 2BS with large effects in both seasons at Kenya. Many *Sr* genes have been mapped on chromosome 2BS, including *Sr19*, *Sr23*, *Sr36*, *Sr39* and *Sr40*. Of these, *Sr19*, *Sr23* and *Sr39* are ineffective against SR. H2B.7 highly likely could be *Sr36* or *Sr40*. Physically, H2B.7 is located around 130–150 Mb proximal to both *Sr36* and *Sr40* and warrants further investigation. On chromosome 3BS, we obtained two haplotypes associated with SR resistance. This chromosome arm harbors *Sr2* gene, one of the most widely used SR resistance genes that has provided durable adult plant rust resistance for over 100 years since its identification. A definitive linkage-pleiotropy test would provide more insight about H3B.1, H3B.6 and *Sr2* gene. An association on chromosome 4DS with large effects in both seasons at Kenya was identified. The only SR resistance gene reported on chromosome 4DS, *Sr41*, is ineffective against the *Ug99* race lineage. The H4D.1 is likely a new locus on 4DS. On chromosome 5BL, two *Sr* genes, *Sr49* and *Sr56*, are located. H5B.22 is 20 and 60 Mb proximal to *Sr49* and *Sr56*, respectively, and thus could highly likely be *Sr49*. H5D.3 is located on chromosome 5DS where no SR resistance genes or QTL have so far been reported. On chromosome 7AL, a minor effect locus H7A.23, reducing disease severity by 6.7 to 12.7% in two Kenya seasons, was identified where two *Sr* genes, *Sr15* and *Sr22*, are located. Whether H7A.23 is *Sr15* or *Sr22* needs further investigation because BLAST results were not helpful in determining the physical distance between H7A.23 and *Sr15* or *Sr22*. The contribution of additive-additive interactions in the genetic architecture of SR resistance has been extensively investigated in wheat using GWAS panels [77–81]. Limited investigations carried out in this area for YR resistance have unveiled from none [82, 83] to little interactions using bi-parental designs [84]. Landrace accessions with different haplotype combinations have been presented in S5 and S6 Tables for GWAM enabled application of the study in wheat breeding.

## Conclusions

The current study is the first investigation of an additive-additive interactions for YR and SR resistance in wheat landrace germplsm set. Over 50% of haplotype-trait associations in this study were found to show an additive-additive interaction effect, which suggests that a network of gene-gene interactions is in part responsible for imparting resistance to both YR and SR in the landrace core set. Specifically, the significant involvement of HBs collocating well known *Sr2* gene in epistatic interactions reinforces that gene(s) in this region impart resistance by both additive and interaction effects. Hot spots of epistatic interactions were found on chromosomes 1BL, 2BL, 5BL and 6AS for YR resistance and on chromosomes 3BS, 7AL and 4DS for and SR resistance, respectively. 73 and 11 accessions with different QTL combinations have been identified for future validation studies by constructing new biparental populations. With further characterization and successful validation, diagnostic markers linked to these resistance genes can be used for breeding wheat varieties with resistance to the wheat rusts.

## Supporting information

S1 Table Correlations among environments for yellow rust and stem rust severity scores. (DOCX)

S2 Table Table presenting the LD Decay in each 21 wheat chromosome. (DOCX)

S3 Table Genomic associations for yellow rust disease in the Mexican bread wheat landraces. (DOCX)

S4 Table Genomic associations for stem rust disease in the Mexican bread wheat landraces. (DOCX)

S5 Table Accessions identified with different YR QTL combinations in Toluca environment for future validation studies. (DOCX)

S6 Table Accessions identified with different SR QTL combinations in Kenya for future validation studies. (DOCX)
