# Supplementary material for: Genome-wide association analysis of Mexican bread wheat landraces for resistance to yellow and stem rust
Source: PLoS One. 2021 Jan 29;16(1):e0246015. doi: 10.1371/journal.pone.0246015 (PMC7846011; doi:10.1371/journal.pone.0246015)
Supplement: S1 Table — (DOCX) [file pone.0246015.s001.docx]

S1 Table: Correlations among environments for yellow rust and stem rust severity scores

1. Pearson’s correlation coefficients between yellow rust reaction of landraces in three year evaluation at Punjab Agriculture University (PAU), Ludhiana, India as well as between Punjab, India and Toluca, Mexico environments.

|  | PAU 2015-16 | PAU 2016-17 | PAU 2017-18 |
| --- | --- | --- | --- |
| PAU 2015-16 | 1 | 0.97629 | 0.96617 |
| PAU 2016-17 | 0.97629 | 1 | 0.98803 |
| PAU 2017-18 | 0.96617 | 0.98803 | 1 |
| Toluca 15 | 0.0383 | 0.04086 | 0.14065 |

PAU 2015-16: Yellow rust evaluation of Mexican wheat landraces at PAU in 2015-16 crop season

PAU 2016-17: Yellow rust evaluation of Mexican wheat landraces at PAU in 2016-17 crop season

PAU 2017-18: Yellow rust evaluation of Mexican wheat landraces at PAU in 2016-17 crop season

Toluca 2015: Yellow rust evaluation of Mexican wheat landraces at Toluca in 2015 crop season

1. Pearson’s correlation coefficients between stem rust reaction of landraces in two year evaluation at Njoro, Kenya (2014-15 and 2015-16).

|  | SRKenya1 | SRKenya2 |
| --- | --- | --- |
| SRKenya1 | 1 | 0.919257 |
| SRKenya2 | 0.919257 | 1 |

Sr Kenya1: Stem rust evaluation of Mexican wheat landraces at Njoro, Kenya in 2014-15 crop season

Sr Kenya1: Stem rust evaluation of Mexican wheat landraces at Njoro, Kenya in 2015-16 crop season

Footnote: Below diagonal values represent correlations values and above diagonal values represent level of significance
