# Supplementary material for: Genome-wide association analysis of Mexican bread wheat landraces for resistance to yellow and stem rust
Source: PLoS One. 2021 Jan 29;16(1):e0246015. doi: 10.1371/journal.pone.0246015 (PMC7846011; doi:10.1371/journal.pone.0246015)
Supplement: S2 Table — (DOCX) [file pone.0246015.s002.docx]

S2 Table. Table presenting the LD Decay in each 21 wheat chromosome.

| Chromosome | LD decay |
| --- | --- |
| 1A | 18Mb |
| 1B | 10Mb |
| 1D | 22Mb |
| 2A | 23Mb |
| 2B | 23Mb |
| 2D | 28Mb |
| 3A | 25Mb |
| 3B | 10Mb |
| 3D | 30Mb |
| 4A | 25Mb |
| 4B | 22Mb |
| 4D* | - |
| 5A | 22Mb |
| 5B | 22Mb |
| 5D | 30Mb |
| 6A | 22Mb |
| 6B | 16Mb |
| 6D | 23Mb |
| 7A | 10Mb |
| 7B | 24Mb |
| 7D | 32Mb |

*Markers were not enough to estimate LD decay.
