# Supplementary material for: Genome-wide association analysis of Mexican bread wheat landraces for resistance to yellow and stem rust
Source: PLoS One. 2021 Jan 29;16(1):e0246015. doi: 10.1371/journal.pone.0246015 (PMC7846011; doi:10.1371/journal.pone.0246015)
Supplement: S3 Table — (DOCX) [file pone.0246015.s003.docx]

S3 Table. Genomic associations for yellow rust disease in the Mexican bread wheat landraces.

| HBs | Chr | Tags | Haplotype based analysis results | | | | | | Single marker-based analysis results | | | | | |
| --- | --- | --- | --- | --- | --- | --- | --- | --- | --- | --- | --- | --- | --- | --- |
| HBs/SNP | Chr | Marker/ Tags | *P*-Value-Toluca | *R^2^*-Toluca | *P*-Value-PAU_16-17 | *R^2^*-PAU_16-17 | *P*-Value-PAU_15-16 | *R*^2^-PAU_15-16 | *P*-Value-Toluca | *R^2^*-Toluca | *P*-Value-PAU_16-17 | *R^2^*-PAU_16-17 | *P*-Value-PAU_15-16 | *R^2^*-PAU_15-16 |
| H1B.11 | 1B | 2276098 |  |  | 6.64E-04 | 0.02 | 1.92E-04 | 0.03 | 2.20E-04 | 0.01 | 1.44E-04 | 0.01 | 2.72E-05 | 0.02 |
| H1B.13 | 1B | 1120791 | 7.54E-04 | 0.02 | 5.17E-04 | 0.02 | 2.77E-04 | 0.02 | 5.59E-05 | 0.02 | 4.71E-05 | 0.01 | 3.07E-05 | 0.02 |
| H1B.17 | 1B | 1090342 | 4.10E-05 | 0.03 | 2.36E-05 | 0.03 | 1.05E-05 | 0.03 | 4.24E-04 | 0.01 | 3.97E-04 | 0.01 | 2.10E-04 | 0.01 |
| H1B.17 | 1B | 984340 |  |  |  |  |  |  | 4.49E-05 | 0.02 | 3.62E-05 | 0.02 | 7.78E-05 | 0.01 |
| H1B.19 | 1B | 999620 | 1.14E-04 | 0.03 | 1.66E-04 | 0.03 | 1.65E-04 | 0.03 | 5.32E-04 | 0.01 | 3.86E-04 | 0.01 | 5.21E-04 | 0.01 |
| H1D.7 | 1D | 2255032 | 1.38E-04 | 0.04 | 1.84E-04 | 0.04 | 3.08E-04 | 0.03 | 4.89E-04 | 0.01 | 5.94E-04 | 0.01 | 2.72E-04 | 0.01 |
| H2B.22 | 2B | 1164349 | 3.32E-05 | 0.03 | 6.95E-05 | 0.03 | 6.19E-05 | 0.03 | 8.46E-07 | 0.02 | 2.88E-06 | 0.02 | 1.97E-06 | 0.02 |
| H2B.46 | 2B | 100373728 | 5.93E-06 | 0.03 | 6.89E-06 | 0.03 | 2.93E-06 | 0.03 | 4.56E-05 | 0.02 | 5.72E-05 | 0.02 | 3.52E-05 | 0.02 |
| H2B.46 | 2B | 1106313 |  |  |  |  |  |  | 1.95E-05 | 0.02 | 2.79E-05 | 0.02 | 2.38E-05 | 0.02 |
| H3A.23 | 3A | 1104683 | 7.92E-05 | 0.03 | 7.94E-05 | 0.03 | 3.03E-05 | 0.03 | 5.17E-06 | 0.02 | 6.64E-06 | 0.02 | 2.62E-06 | 0.02 |
| H4A.11 | 4A | 100403792 |  |  | 6.83E-04 | 0.02 | 3.16E-04 | 0.03 | 3.77E-05 | 0.02 | 2.14E-05 | 0.02 | 1.64E-05 | 0.02 |
| H4B.19 | 4B | 1090994 | 2.46E-04 | 0.02 | 3.46E-04 | 0.02 | 1.43E-04 | 0.03 | 7.29E-05 | 0.01 | 5.32E-05 | 0.02 | 4.50E-05 | 0.02 |
| H6A.6 | 6A | 1094663 | 7.05E-04 | 0.02 | 8.95E-04 | 0.02 | 5.46E-04 | 0.02 | 1.27E-04 | 0.02 | 1.59E-04 | 0.02 | 8.00E-05 | 0.02 |
| H6A.7 | 6A | 1219443 | 5.37E-07 | 0.04 | 5.87E-07 | 0.04 | 1.86E-07 | 0.04 | 1.28E-04 | 0.01 | 1.68E-04 | 0.01 | 7.49E-05 | 0.02 |
| H6A.7 | 6A | 1695233 |  |  |  |  |  |  | 2.80E-06 | 0.02 | 6.48E-06 | 0.02 | 6.44E-06 | 0.02 |

Footnote: Below diagonal values represent correlations values and above diagonal values represent level of significance
