# Supplementary material for: Genome-wide association analysis of Mexican bread wheat landraces for resistance to yellow and stem rust
Source: PLoS One. 2021 Jan 29;16(1):e0246015. doi: 10.1371/journal.pone.0246015 (PMC7846011; doi:10.1371/journal.pone.0246015)
Supplement: S4 Table — (DOCX) [file pone.0246015.s004.docx]

S4 Table. Genomic associations for stem rust disease in the Mexican bread wheat landraces.

| HBs/SNP | Marker/ Tags | Chr | SMA-P-Value_Kn1 | SMA-*R^2^*_Kn1 | SMA-P-Value_Kn2 | SMA-*R^2^*_Kn2 | HB-P-Value_Kn1 | HB-*R^2^*_Kn1 | HB-P-Value_Kn2 | HB-*R^2^*_Kn2 |
| --- | --- | --- | --- | --- | --- | --- | --- | --- | --- | --- |
| H1A.36 | 1126203 | 1A | 6.15E-05 | 0.01362 | 8.57E-12 | 0.05015 | 3.15E-04 | 0.02108 | 1.99E-09 | 0.05404 |
| H2A.1 | 1232836 | 2A | 4.12E-07 | 0.02032 | 4.87E-06 | 0.02147 | 6.87E-05 | 0.02538 | 2.27E-04 | 0.03007 |
| H2A.13 | 1E+08 | 2A | 6.86E-05 | 0.01301 | 5.13E-12 | 0.05024 | 7.84E-05 | 0.02514 | 1.16E-11 | 0.06725 |
| H2B.17 | 2277182 | 2B | 9.66E-07 | 0.01888 | 9.82E-07 | 0.02457 | 3.60E-05 | 0.02342 | 1.43E-05 | 0.03248 |
| H2B.24 | 1E+08 | 2B | 1.19E-06 | 0.02209 | 3.45E-06 | 0.02652 | 1.91E-05 | 0.03631 | 3.43E-11 | 0.07828 |
| H2B.39 | 1E+08 | 2B | 0.000448 | 0.01177 | 4.67E-05 | 0.0204 | 8.85E-05 | 0.0332 | 1.52E-04 | 0.04164 |
| H2B.46 | 1E+08 | 2B | 0.000633 | 0.01071 | 7.09E-06 | 0.02353 | 1.25E-06 | 0.0309 | 1.62E-07 | 0.04459 |
| H2D.8 | 1E+08 | 2D | 5.4E-06 | 0.01754 | 3.95E-05 | 0.0186 | 6.85E-05 | 0.03373 | 9.00E-15 | 0.09597 |
| H3A.3 | 1220431 | 3A | 0.000914 | 0.00892 | 4.82E-16 | 0.06733 | 2.63E-06 | 0.03126 | 2.40E-14 | 0.07989 |
| SNP | 2280990 | 3A | 2.53E-06 | 0.02206 | 2.24E-05 | 0.02325 |  |  |  |  |
| H3A.4 | 1E+08 | 3A | 2.17E-07 | 0.02127 | 7.41E-09 | 0.03429 | 1.41E-05 | 0.02333 | 5.53E-10 | 0.05165 |
| H3A.5 | 1E+08 | 3A | 1.7E-09 | 0.02919 | 6.03E-09 | 0.03535 | 6.35E-09 | 0.05137 | 6.51E-07 | 0.05556 |
| H3A.6 | 3023460 | 3A | 7.51E-07 | 0.02522 | 2.74E-07 | 0.034 | 4.32E-06 | 0.03038 | 1.22E-06 | 0.04229 |
| SNP | 1066502 | 3A | 1.01E-08 | 0.02784 | 2.45E-08 | 0.03409 |  |  |  |  |
| H3A.7 | 982358 | 3A | 1.65E-09 | 0.0306 | 2.31E-09 | 0.03918 | 2.27E-07 | 0.03553 | 7.76E-07 | 0.04331 |
| SNP | 1095938 | 3A | 3.72E-08 | 0.02676 | 1.61E-08 | 0.03329 |  |  |  |  |
| H3A.12 | 1208317 | 3A | 0.000126 | 0.01187 | 7.74E-08 | 0.02999 | 9.31E-04 | 0.01905 | 1.85E-12 | 0.06853 |
| H3B.1 | 1196897 | 3B | 3.15E-05 | 0.01639 | 4.99E-06 | 0.02547 | 1.26E-04 | 0.02276 | 2.89E-04 | 0.02759 |
| H3B.6 | 1E+08 | 3B | 3.16E-05 | 0.01717 | 0.000122 | 0.01919 | 3.62E-05 | 0.02501 | 4.89E-04 | 0.02632 |
| SNP | 3064675 | 3B | 4.76E-06 | 0.01687 | 1.29E-05 | 0.01986 |  |  |  |  |
| H3B.17 | 1E+08 | 3B | 9.88E-05 | 0.01285 | 1.3E-05 | 0.02084 | 7.91E-04 | 0.02077 | 2.97E-04 | 0.02941 |
| H3D.1 | 1E+08 | 3D | 1.41E-05 | 0.016 | 0.000277 | 0.01436 | 5.43E-06 | 0.03879 | 4.28E-09 | 0.06743 |
| SNP | 1E+08 | 3D | 4.81E-11 | 0.0404 | 4.61E-10 | 0.04775 |  |  |  |  |
| H4A.18 | 2303516 | 4A | 0.000543 | 0.01046 | 3.5E-05 | 0.01968 | 8.85E-04 | 0.02055 | 5.49E-05 | 0.03348 |
| H4B.4 | 2254072 | 4B | 0.000922 | 0.01043 | 3.77E-12 | 0.05787 | 5.72E-06 | 0.02827 | 1.11E-10 | 0.06011 |
| H4B.18 | 986417 | 4B | 2.86E-08 | 0.02629 | 1.53E-05 | 0.02104 | 2.20E-06 | 0.02993 | 3.14E-04 | 0.02739 |
| H4D.1 | 1089593 | 4D | 1.72E-10 | 0.03194 | 9.13E-15 | 0.06055 | 1.36E-09 | 0.04044 | 4.01E-13 | 0.06899 |
| H5A.31 | 1002728 | 5A | 1.41E-06 | 0.01934 | 4.94E-10 | 0.04133 | 4.88E-04 | 0.02027 | 2.61E-09 | 0.05347 |
| H5B.10 | 1061363 | 5B | 2.88E-05 | 0.01504 | 1.72E-08 | 0.03461 | 3.13E-04 | 0.03055 | 1.70E-05 | 0.0474 |
| H5B.14 | 2241527 | 5B | 1.22E-09 | 0.0295 | 8.14E-07 | 0.02541 | 1.19E-08 | 0.03691 | 1.16E-07 | 0.04299 |
| H5B.17 | 1166904 | 5B | 1.76E-08 | 0.02675 | 0.000103 | 0.0166 | 3.76E-07 | 0.03465 | 5.46E-09 | 0.05422 |
| H5B.22 | 1126798 | 5B | 5.98E-05 | 0.01738 | 4.03E-09 | 0.04569 | 4.31E-06 | 0.03924 | 1.09E-11 | 0.07869 |
| H6A.16 | 984529 | 6A | 0.000771 | 0.012 | 0.000615 | 0.01588 | 4.72E-07 | 0.03426 | 5.71E-07 | 0.044 |
| SNP | 3027201 | 6A | 3.97E-05 | 0.01613 | 7.14E-08 | 0.03582 |  |  |  |  |
| H7A.14 | 1008985 | 7A | 7.47E-07 | 0.01924 | 1.77E-07 | 0.02764 | 9.52E-06 | 0.02572 | 1.20E-05 | 0.03287 |
| SNP | 1099321 | 7A | 9.7E-07 | 0.01937 | 6.52E-07 | 0.02592 |  |  |  |  |
| H7A.19 | 1008941 | 7A | 2.13E-05 | 0.01568 | 3.43E-09 | 0.03872 | 3.57E-04 | 0.02229 | 6.38E-07 | 0.04375 |
| H7A.23 | 2262174 | 7A | 5.08E-07 | 0.02246 | 1.11E-13 | 0.06198 | 7.04E-05 | 0.02533 | 4.06E-12 | 0.06942 |
| SNP | 988655 | 7A | 4.77E-05 | 0.01396 | 1.66E-05 | 0.02026 |  |  |  |  |
| H7A.28 | 3064818 | 7A | 8.06E-07 | 0.0201 | 4.2E-05 | 0.0173 | 2.86E-08 | 0.03547 | 8.18E-04 | 0.02319 |
| H7A.30 | 997971 | 7A | 4.35E-06 | 0.02027 | 4.49E-10 | 0.04899 | 7.90E-06 | 0.0277 | 2.18E-08 | 0.04894 |
| H7A.35 | 1233998 | 7A | 0.000107 | 0.01436 | 5.44E-09 | 0.03916 | 3.01E-04 | 0.02262 | 9.12E-10 | 0.05569 |
| SNP | 1E+08 | 7A | 1.41E-05 | 0.02091 | 7.64E-10 | 0.05118 |  |  |  |  |
| H7B.6 | 1136152 | 7B | 0.000186 | 0.01213 | 3.25E-07 | 0.02963 | 1.44E-04 | 0.024 | 3.95E-11 | 0.06469 |
| H7D.4 | 1074330 | 7D | 1.88E-08 | 0.02538 | 0.000235 | 0.01426 | 2.99E-05 | 0.03541 | 1.33E-04 | 0.042 |
