# Supplementary material for: Genome-wide association analysis of Mexican bread wheat landraces for resistance to yellow and stem rust
Source: PLoS One. 2021 Jan 29;16(1):e0246015. doi: 10.1371/journal.pone.0246015 (PMC7846011; doi:10.1371/journal.pone.0246015)
Supplement: S5 Table — (DOCX) [file pone.0246015.s005.docx]

S5 Table. Accessions identified with different YR QTL combinations in Toluca environment for future validation studies.

| GID | H3B.34 | H5B.22 | H1A.24 | H2B.32 | H5B.19 | H1A.33 | H1B.19 | Mean YR severity scores-Toluca |
| --- | --- | --- | --- | --- | --- | --- | --- | --- |
| 162361 | AA | GGT | CNG | GT | CG | NC | CC | 60 |
| 192313 | AA | GGT | GNG | AT | CG | GT | CC | 20 |
| 162095 | GA | CGC | CTG | GT | CG | GT | CC | 20 |
| 221651 | GG | CGC | GCG | AT | CG | GT | CC | 40 |
| 162286 | GG | CGC | CCG | AT | CN | GT | CC | 50 |
| 207077 | GN | GGT | CCA | GT | CG | GT | CC | 10 |
| 157844 | GG | GGT | CCA | GT | CG | GN | CC | 50 |
| 158653 | GG | GGT | CCA | AT | CG | GT | CC | 10 |
| 159110 | GA | GGT | CCA | GT | CG | NC | CC | 30 |
| 159134 | GA | GGT | CCA | GT | CG | AN | CC | 40 |
| 193427 | GA | GGT | CCA | GT | CG | GT | CC | 50 |
| 193558 | GA | GGT | CCA | GT | CG | GT | CC | 40 |
| 193573 | GA | GGN | CCA | GT | CN | GT | CC | 40 |
| 192328 | GA | GGT | CCA | GT | CG | GT | CC | 30 |
| 193868 | GG | GGT | CNG | AC | CN | GT | CC | 50 |
| 194285 | GA | GGT | NCG | AC | CG | GT | CC | 50 |
| 194907 | GG | GGT | CCG | AC | CG | GT | CC | 10 |
| 194909 | GG | GGT | CCG | AC | CN | GT | CC | 30 |
| 191161 | GG | GGT | NCG | AC | CG | GT | CC | 10 |
| 191777 | GG | GGN | CCG | AC | CG | GT | CC | 30 |
| 192299 | GG | GGT | CCG | AC | CG | GT | CC | 20 |
| 158771 | GG | NGT | GCG | AC | CG | GT | CC | 20 |
| 162023 | GA | GGT | CCG | AC | CG | GT | CC | 40 |
| 207080 | GG | GGT | NCG | AC | CG | NC | CC | 20 |
| 158113 | GG | GGT | CCG | AC | CG | GT | CC | 20 |
| 217508 | GG | GGN | GCG | AC | CG | GT | CC | 20 |
| 225585 | AN | GGT | CNG | AC | CG | GT | CC | 30 |
| 320331 | GA | GGT | CCG | AC | CG | NT | CC | 30 |
| 320333 | GA | GGT | CCG | AC | CG | GT | CC | 20 |
| 320583 | GA | GGT | GNG | GT | CA | NC | CC | 40 |
| 206896 | GG | GGT | GCG | GT | CG | AC | CC | 50 |
| 158881 | GA | GGT | GCG | GT | CG | AC | CC | 40 |
| 195884 | GA | GGT | GNG | GT | CG | AC | CC | 60 |
| 225359 | NA | NGT | GCG | AN | CN | AC | CC | 20 |
| 225573 | GG | GGT | NCG | AT | CG | AC | CC | 10 |
| 225673 | GG | GGT | GCG | AT | NG | AC | CC | 10 |
| 225676 | GG | GGT | GCG | NT | CG | AC | CC | 30 |
| 225738 | GG | GGT | GCG | AN | CG | AC | CC | 40 |
| 320566 | GA | GGT | GNG | GT | NA | AC | CC | 30 |
| 320309 | GG | GGT | CCG | AT | CN | GT | CC | 60 |

Footnote: The presence of QTL/QTL combination is shown as green colored cells
