# Supplementary material for: Genome-wide association analysis of Mexican bread wheat landraces for resistance to yellow and stem rust
Source: PLoS One. 2021 Jan 29;16(1):e0246015. doi: 10.1371/journal.pone.0246015 (PMC7846011; doi:10.1371/journal.pone.0246015)
Supplement: S6 Table — (DOCX) [file pone.0246015.s006.docx]

S6 Table. Accessions identified with different SR QTL combinations in Kenya for future validation studies.

| GID | H2A.13 | 2B.7 | H3B.1 | H5B.22 | H5D.3 | H3B.1+H2A.13 | H3B.1+H5D.3 | H3B.1+H7A.23 | Mean SR severity scores |
| --- | --- | --- | --- | --- | --- | --- | --- | --- | --- |
| 159089 |  |  |  |  |  |  |  |  | 50 |
| 159132 |  |  |  |  |  |  |  |  | 45 |
| 266700 |  |  |  |  |  |  |  |  | 40 |
| 193648 |  |  |  |  |  |  |  |  | 55 |
| 193294 |  |  |  |  |  |  |  |  | 80 |
| 193271 |  |  |  |  |  |  |  |  | 55 |
| 191914 |  |  |  |  |  |  |  |  | 60 |
| 159133 |  |  |  |  |  |  |  |  | 45 |
| 191118 |  |  |  |  |  |  |  |  | 40 |
| 191723 |  |  |  |  |  |  |  |  | 35 |
| 194989 |  |  |  |  |  |  |  |  | 30 |

Footnote: The presence of QTL/QTL combination is shown as green colored cells
